# Supplementary material for: Splice-Junction-Based Mapping of Alternative Isoforms in the Human Proteome
Source: Cell Rep. Author manuscript; Available in PMC 2020 Jan 15. (PMC6961840; doi:10.1016/j.celrep.2019.11.026)
Supplement: 2 [file NIHMS1546469-supplement-2.zip › DF1/PXD006675/PulmonaryArtery/PulmonaryArtery_20_ELN_AQLLPGALAAAK.pdf]

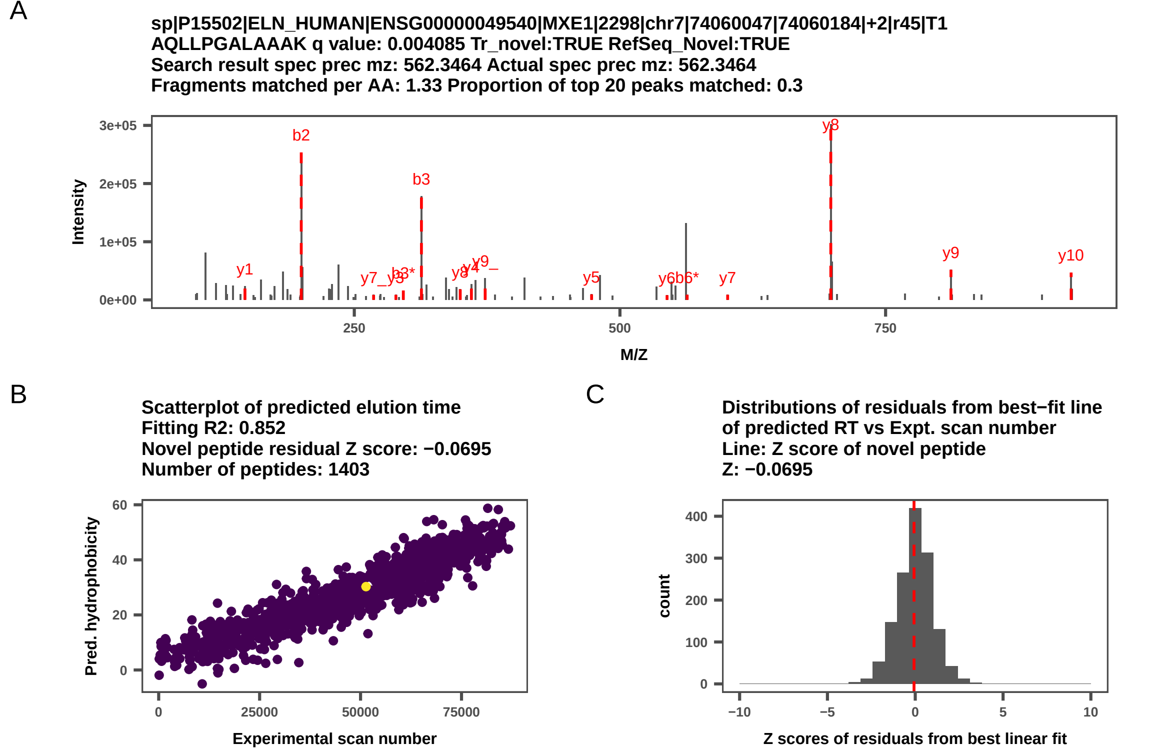
A
sp|P15502|ELN_HUMAN|ENSG00000049540|MXE1|2298|chr7|74060047|74060184|+2|r45|T1
AQLLPGALAAAK q value: 0.004085 Tr_novel:TRUE RefSeq_Novel:TRUE
Search result spec prec mz: 562.3464 Actual spec prec mz: 562.3464
Fragments matched per AA: 1.33 Proportion of top 20 peaks matched: 0.3
Intensity
3e+05
2e+05
1e+05
0e+00
b2
b3
y8
y9
y10
y1
y5
y7
250
500
750
M/Z
B
Scatterplot of predicted elution time
Fitting R2: 0.852
Novel peptide residual Z score: −0.0695
Number of peptides: 1403
Pred. hydrophobicity
60
40
20
0
25000
50000
75000
Experimental scan number
C
Distributions of residuals from best−fit line
of predicted RT vs Expt. scan number
Line: Z score of novel peptide
Z: −0.0695
count
400
300
200
100
0
−10
−5
5
10
Z scores of residuals from best linear fit
